# Nef variants from non-pathogenic lentiviral strains inhibit iron uptake through an AP2-dependent inhibition of transferrin endocytosis

**DOI:** 10.1186/1742-4690-10-S1-P42

**Published:** 2013-09-19

**Authors:** Herwig Koppensteiner, Kristin Höhne, Marcos Vinicius Gondim, Francois-Xavier Gobert, Miriam Widder, Swantje Gundlach, Anke Heigele, Frank Kirchhoff, Michael Winkler, Philippe Benaroch, Michael Schindler

**Affiliations:** 1 Institute of Virology, Helmholtz Zentrum Munich, German Research Center for Environmental Health, Munich, Germany; 2 Heinrich Pette Institute, Leibniz Institute for Experimental Virology, Hamburg, Germany; 3 Institut Curie, INSERM U932, Paris, France; 4 Ulm University Medical Center, Institute of Molecular Virology, Ulm, Germany; 5 German Primate Center, Göttingen, Germany

## Background

Increased cellular iron levels are associated with high mortality in HIV-1 infection. Moreover iron is an important cofactor for viral replication, raising the question of whether modulation of intracellular iron can be linked to the pathogenicity of lentiviral infections. Here, we evaluated the effect on cellular iron uptake upon expression of the accessory protein Nef from divergent lentiviruses.

## Results

Surface Transferrin receptor (TfR) levels are unaffected by Nef proteins of HIV-1 and its simian precursors but elevated in cells expressing Nefs from most other primate lentiviruses due to reduced TfR internalization. The SIV Nef-mediated reduction of TfR endocytosis is dependent on an N-terminal AP2 binding motif that is not required for downmodulation of CD4, CD28, CD3 or MHCI. Importantly, SIV Nef-induced inhibition of TfR endocytosis leads to the reduction of Transferrin uptake and intracellular iron concentration and is accompanied by attenuated lentiviral replication in macrophages.

## Conclusions

Thus, this new SIV Nef function might limit viral replication in myeloid cells and may contribute to the absence of disease in SIV-infected monkeys. Altogether, lentiviruses actively modulate replication by the manipulation of cellular iron, which is an important determinant for viral pathogenicity.

